# The New State Health Officer

**Published:** 1901-11

**Authors:** 


THE NEW STATE HEALTH OFFICER.—Dr. Geo. R. Tabor, of Bryan, has been appointed State Health Officer *vice* Dr. W. F. Blunt, resigned.

This is a most excellent appointment, and the JOURNAL congratulates the Governor upon so judicious a selection. Indeed, he and the State are to be congratulated upon securing the services of so capable and popular a man; for, the choice of a State health officer being limited by law to immunes, and to physicians identified with public sanitation, is very narrow; and there are few physicians in the State eligible under the law, and qualified to fill the position, who can afford to break up, leave home and take the place for a salary largely disproportioned to the work involved and the special qualifications needed, and that, too, for two years.

Dr. Tabor is a strong, healthy young man, not yet forty. He is a representative of the better class of the younger physicians, and is very popular. His appointment, I am sure, will have the endorsement of the profession at large, and he will have the confidence and support of his colleagues throughout the State.

Dr. Tabor was raised in Bryan, and has lived there all his life. He has been County Health Officer of Brazos county many years, and has taken an active part in the agitation for sanitary progress and reform in Texas. He is a graduate of the Louisville Medical College, class of 1888. Dr. Tabor removed his family to Austin and took charge of the office November 1st, inst.

